# Commentary: Improvement in diagnostic-therapeutic care pathways for women with migraine: an Italian Delphi panel

**DOI:** 10.3389/fneur.2024.1507261

**Published:** 2024-11-20

**Authors:** Alessandro Viganò, Paola Tiberio, Nicholas Diani, Alberto Zambelli, Armando Santoro, Rita De Sanctis

**Affiliations:** ^1^IRCCS Fondazione Don Carlo Gnocchi, Milan, Italy; ^2^Department of Biomedical Sciences, Humanitas University, Pieve Emanuele, MI, Italy; ^3^Medical Oncology and Hematology Unit, IRCCS Humanitas Research Hospital, Rozzano, MI, Italy

**Keywords:** migraine, woman, Delphi, gynecologist, neurologist, pediatrician, oncologist, pharmacist

## 1 Introduction

The expert opinion paper “Improvement in diagnostic-therapeutic care pathways for women with migraine: an Italian Delphi panel” by Cevoli et al. (1) presents indications for improving diagnostic and therapeutic pathways for women suffering from migraine, addressing both old and emerging health conditions. We sincerely praised their work for addressing the important issue of breast cancer (BC) in women's health. Given the high prevalence of both migraine and BC in the global population (2–5), understanding how these conditions interact with each other is crucial. In this commentary, we expand on some of the panel's themes and provide some additional insights that may impact headache management in cancer patients, suggesting areas for further exploration.

## 2 Headache prevalence in breast cancer patients

Migraine prevalence seems higher in women with BC compared to the global female population. Previous research, mainly survey and retrospective studies, could not definitively conclude whether migraine was protective, neutral or detrimental regarding BC diagnosis and prognosis (6–12). To fill this gap, we studied a large cohort of BC patients to investigate actual prevalence and changes of headache at BC diagnosis, during treatments and follow-up (13). We found migraine prevalence in BC patients to be 56.1% compared to 17% in the general female population, suggesting either shared pathophysiological mechanisms or a statistical coincidence, warranting further investigation.

## 3 Headache during breast cancer: are the two conditions related or independent?

The association between headache and a systemic condition always raises questions about whether it is a secondary symptom or an exacerbation of a primary headache. Presently, there is no clinical data about the effectiveness of migraine medications in primary migraine in patients with concomitant BC diagnosis. As a surrogate of direct evidence, we run a [18F]-FDG PET/CT study of the brain metabolic state in newly diagnosed BC patients undergoing neoadjuvant chemotherapy (14). We divided these patients in three groups (migraine, tension-type headache [TTH] and non-headache) according strict application of ICHD3 criteria (15) and studied these patients with [18F]-FDG PET/CT before and after the chemotherapy course.

While migraine patients did not exhibit significant metabolic changes before and after chemotherapy, TTH patients showed hypometabolism in the right insula and temporal lobe, regions implicated in pain processing and emotional regulation, which appeared to be modified by chemotherapy and to correlate with tumor [18F]-FDG PET/CT metabolism. This suggests a potential link between TTH and BC metabolism, which may be mediated by the interaction between systemic chemotherapy and brain activity. Therefore, it seems plausible that, while TTH-like headache could represent an epiphenomenon of BC, a headache with migraine features represents more an actual comorbidity than a symptom of BC.

The picture of the intertwining between migraine and BC is also complicated by possible cofounders as migraine medications. The use of preventive and acute migraine medications may influence the risk of developing BC later in life, though it is not supported by epidemiological evidence (7). In fact, although some inconsistencies across different studies emerged, it has been demonstrated that certain anti-migraine treatments may cause alteration in the level of sex hormones and prolactin (16), which in turn has been suggested to induce BC cell proliferation and spread and to be associated with increased BC risk (17–20). For instance, the use of amitriptyline and valproate (the latter nowadays less commonly utilized in women of childbearing age) for migraine prevention, or the association of metoclopramide for the control of acute migraine attacks, may alter prolactin levels. However, further complicating the scenario, metoclopramide is also used for chemotherapy-induced nausea and vomiting (CINV) (21). While prolonged metoclopramide use has not been definitively linked to increased BC risk, this remains an interpretative challenge. Prolactin testing is not routinely included in BC or migraine follow-up protocols, potentially overlooking a key confounder. Thus, further prospective cohort and translational studies investing the risk of BC development in patients with hyperprolactinemia induced by anti-migraine medications are definitely needed.

## 4 Oncologist-neurologist relationship

Another critical point regards the communication between oncologists and neurologists to improve the management of these patients. We believe educational programs can facilitate referrals of BC patients to headache-specialized centers. In a pilot study of 38 BC patients with headache, only 2 patients accepted a neurologist referral, while 36 declined stating that they were too tied to their cancer management schedules to focus on other health issues (22). The main barriers appear to be time constrains and disillusionment. A possible way to overcome this problem could be to implement patient empowerment programs to either treat the pain directly (as with pain education) (23–25) or to restore patients' confidence in headache treatment. Specialized facilities with dedicated slots could be of help address this challenge.

## 5 Migraine headache worsened during antineoplastic treatments

The Delphi panel also highlighted the potential impact of systemic therapies on headache frequency. When dealing with headache, and migraine in particular, one key point is to identify triggers or secondary causes and exclude them before starting any preventive therapy. In BC patients, antineoplastic medications could in theory have a detrimental effect on migraine pain, especially for anti-estrogens treatments in patients with luminal BC (1). However, on the other hand, inducing a pharmacological menopausal state, anti-hormone therapies could have little or no impact on the development of BC (26). On the other hand, in our cohort of 440 BC patients, we have found that local radiotherapy worsened headaches, especially in migraine with aura, while other treatments didn't affect headache status (13). Moreover, migraine patients were more prone to developing peripheral neuropathies after taxane-based chemotherapy than patients without headache. Although these data need to be confirmed, they could orient specialists to check for migraine onset or worsening in patients undergoing radiotherapy.

## 6 Discussion

The Delphi consensus by Cevoli et al. on optimizing diagnostic-therapeutic pathways for women with migraine represents a major contribution. In this comment, we shared findings from prospective studies on BC patients, suggesting that TTH may be more closely linked to BC and fluctuate during BC treatments, while migraine appears to remain independent of BC diagnosis or chemotherapy. To date the only oncological treatment that had an impact on migraine was local radiotherapy, although a clear explanation of that is still missing.

We also agree with the panel's emphasis on emotional distress in headache management, as BC patients are particularly vulnerable to emotional stress due to their cancer diagnosis and treatment. In our study, despite the high prevalence of headaches, very few BC patients expressed interest in neurology referral for headache management. This reluctance may stem from a perceived normalization of headache symptoms in the context of cancer care, as well as the prioritization of oncological treatment over other health concerns. It underscores the need for better patient education and a multidisciplinary approach to headache management in cancer patients, incorporating not only oncological care but also neurology and mental health support.

In conclusion, as we continue to explore the complex interplay between headache and cancer, a multidisciplinary approach will be essential for optimizing patient care. The diagnostic-therapeutic pathways proposed by Cevoli et al. provide a valuable framework for improving migraine management. We look forward to seeing further research in this area that will continue to improve the quality of care for women with primary headaches in the context of comorbid conditions like BC.

## References

[1] Cevoli S Barbanti P Finocchi C Benedan L Mariani P Orthmann N. Improvement in diagnostic-therapeutic care pathways for women with migraine: an Italian Delphi panel. Front Neurol. (2024) 15:1436258. 10.3389/fneur.2024.1436258 39301474 PMC11412109

[2] Bray F Laversanne M Sung H Ferlay J Siegel RL Soerjomataram I. Global cancer statistics 2022: GLOBOCAN estimates of incidence and mortality worldwide for 36 cancers in 185 countries. CA Cancer J Clin. (2024) 74:229–63. 10.3322/caac.21834 38572751

[3] European Network of Cancer Registries. Available online: https://www.encr.eu (accessed September 15, 2024).

[4] Vos T Flaxman AD Naghavi M Lozano R Michaud C Ezzati M. Years lived with disability (YLDs) for 1160 sequelae of 289 diseases and injuries 1990-2010: a systematic analysis for the Global Burden of Disease Study 2010. Lancet. (2012) 380:2163–96. 10.1016/S0140-6736(12)61729-2 23245607 PMC6350784

[5] GBD 2016 Headache Collaborators. Global, regional, and national burden of migraine and tension-type headache, 1990-2016: a systematic analysis for the Global Burden of Disease Study 2016. Lancet Neurol. (2018) 17:954–76. 10.1016/S1474-4422(18)30322-3 30353868 PMC6191530

[6] Peng C Wu K Chen X Lang H Li C He L. Migraine and risk of breast cancer: a systematic review and meta-analysis. Clin Breast Cancer. (2023) 23:e122–30. 10.1016/j.clbc.2022.12.011 36624014

[7] Wu X Wang M Li S Zhang Y. Migraine and breast cancer risk: a meta-analysis of observational studies based on MOOSE compliant. Medicine (Baltimore). (2016) 95:e4031. 10.1097/MD.0000000000004031 27472675 PMC5265812

[8] Hesari E Ahmadinezhad M Arshadi M Azizi H Khodamoradi F. The association between migraine and breast cancer risk: a systematic review and meta-analysis. PLoS ONE. (2022) 17:e0263628. 10.1371/journal.pone.0263628 35143585 PMC8830615

[9] Winter AC Rice MS Fortner RT Eliassen AH Kurth T Tamimi RM. Migraine and breast cancer risk: a prospective cohort study and meta-analysis. J Natl Cancer Inst. (2014) 107:381. 10.1093/jnci/dju381 25505231 PMC4296198

[10] Fan CY Lin CS Huang WY Lin KT Chao HL Tsao CC. Association between migraine and breast cancer risk: a population-based cohort study and literature review. J Womens Health (Larchmt). (2018) 27:1499–507. 10.1089/jwh.2018.6929 30183462

[11] Rezaeian S Veisani Y Ghorbani M Delpisheh A Abbastabar H. Migraine history and breast cancer risk: a systematic review and meta-analysis. Adv Breast Cancer Res. (2015) 4:63–70. 10.4236/abcr.2015.43007

[12] Tiberio P Viganò A Ilieva MB Pindilli S Bianchi A Zambelli A. The role of female reproductive hormones in the association between migraine and breast cancer: an unanswered question. Biomedicines. (2023) 11:1613. 10.3390/biomedicines11061613 37371707 PMC10295631

[13] Ilieva MB Tiberio P Torrisi R Lanzone J Di Piero V Santoro A. Profiling the spectrum of headache disorders on 440 breast cancer patients: highlights on clinical and pathological mechanisms. Biomedicines. (2023) 11:1059. 10.3390/biomedicines11041059 37189678 PMC10135877

[14] Antunovic L Artesani A Viganò A Chiti A Santoro A Sollini M. Imaging correlates between headache and breast cancer: an [18F]FDG PET study. Cancers (Basel). (2023) 15:4147. 10.3390/cancers15164147 37627174 PMC10453040

[15] Arnold M. Headache classification committee of the international headache society (IHS) the international classification of headache disorders, 3rd edition. Cephalalgia. (2018) 38:1–211. 10.1177/0333102417738202 29368949

[16] Szewczyk AK Ulutas S Aktürk T Al-Hassany L Börner C Cernigliaro F. Prolactin and oxytocin: potential targets for migraine treatment. J Headache Pain. (2023) 24:31. 10.1186/s10194-023-01557-6 36967387 PMC10041814

[17] Clevenger CV Furth PA Hankinson SE Schuler LA. The role of prolactin in mammary carcinoma. Endocr Rev. (2003) 24:1–27. 10.1210/er.2001-0036 12588805 PMC1698952

[18] Johnston AN Bu W Hein S Garcia S Camacho L Xue L. Hyperprolactinemia-inducing antipsychotics increase breast cancer risk by activating JAK-STAT5 in precancerous lesions. Breast Cancer Res. (2018) 20:42. 10.1186/s13058-018-0969-z 29778097 PMC5960176

[19] Tworoger SS Eliassen AH Sluss P Hankinson SE. A prospective study of plasma prolactin concentrations and risk of premenopausal and postmenopausal breast cancer. J Clin Oncol. (2007) 25:1482–8. 10.1200/JCO.2006.07.6356 17372279

[20] Tworoger SS Sluss P Hankinson SE. Association between plasma prolactin concentrations and risk of breast cancer among predominately premenopausal women. Cancer Res. (2006) 66:2476–82. 10.1158/0008-5472.CAN-05-3369 16489055

[21] Molitch ME. Medication-induced hyperprolactinemia. Mayo Clin Proc. (2005) 80:1050–7. 10.4065/80.8.1050 16092584

[22] De Sanctis R Viganò A Pindilli S Torrisi R Santoro A. A pilot analysis of headache disorders in breast cancer patients. Neurol Sci. (2022) 43:3313–20. 10.1007/s10072-021-05698-x 34817729

[23] Balordi M Tiberio P Castaldo M Viganò A Jacobs F Zambelli A. Empowering beyond pain: pain neuroscience education interventions in breast cancer survivorship care. Cancers (Basel). (2024) 16:2806. 10.3390/cancers16162806 39199580 PMC11353171

[24] González-Martín AM Aguilera-García I Castellote-Caballero Y Rivas-Campo Y Bernal-Suárez A Aibar-Almazán A. Effectiveness of therapeutic education in patients with cancer pain: systematic review and meta-analysis. Cancers (Basel). (2023) 15:4123. 10.3390/cancers15164123 37627151 PMC10452673

[25] De Groef A Meeus M Heathcote LC Wiles L Catley M Vogelzang A. Treating persistent pain after breast cancer: practice gaps and future directions. J Cancer Surviv. (2023) 17:1698–707. 10.1007/s11764-022-01194-z 35275361 PMC8914454

[26] van Lohuizen R Paungarttner J Lampl C MaassenVanDenBrink A Al-Hassany L. Considerations for hormonal therapy in migraine patients: a critical review of current practice. Expert Rev Neurother. (2023) 24:1–21. 10.1080/14737175.2023.2296610 38112066 PMC10791067

